# The Role of Interferon Regulatory Factors in Liver Diseases

**DOI:** 10.3390/ijms25136874

**Published:** 2024-06-22

**Authors:** Chuanfei Zeng, Xiaoqin Zhu, Huan Li, Ziyin Huang, Mingkai Chen

**Affiliations:** Department of Gastroenterology, Renmin Hospital of Wuhan University, No. 99 Zhang Zhidong Road, Wuhan 430060, China; zengcf2429@whu.edu.cn (C.Z.); zhuxq999@163.com (X.Z.); 2022203020033@whu.edu.cn (H.L.); ziyin0922@163.com (Z.H.)

**Keywords:** interferon regulatory factors, hepatic ischemia-reperfusion injury, liver injury, nonalcoholic fatty liver disease, cirrhosis, hepatocellular carcinoma

## Abstract

The interferon regulatory factors (IRFs) family comprises 11 members that are involved in various biological processes such as antiviral defense, cell proliferation regulation, differentiation, and apoptosis. Recent studies have highlighted the roles of IRF1-9 in a range of liver diseases, including hepatic ischemia–reperfusion injury (IRI), alcohol-induced liver injury, Con A-induced liver injury, nonalcoholic fatty liver disease (NAFLD), cirrhosis, and hepatocellular carcinoma (HCC). IRF1 is involved in the progression of hepatic IRI through signaling pathways such as PIAS1/NFATc1/HDAC1/IRF1/p38 MAPK and IRF1/JNK. The regulation of downstream IL-12, IL-15, p21, p38, HMGB1, JNK, Beclin1, β-catenin, caspase 3, caspase 8, IFN-γ, IFN-β and other genes are involved in the progression of hepatic IRI, and in the development of HCC through the regulation of PD-L1, IL-6, IL-8, CXCL1, CXCL10, and CXCR3. In addition, IRF3-PPP2R1B and IRF4-FSTL1-DIP2A/CD14 pathways are involved in the development of NAFLD. Other members of the IRF family also play moderately important functions in different liver diseases. Therefore, given the significance of IRFs in liver diseases and the lack of a comprehensive compilation of their molecular mechanisms in different liver diseases, this review is dedicated to exploring the molecular mechanisms of IRFs in various liver diseases.

## 1. Introduction

Interferon regulatory factors (IRFs) are a family of transcription factors that play crucial roles in various aspects of the immune response, including the development and differentiation of immune cells, as well as the regulation of responses to pathogens [1]. IRF1, the first identified IRF that induced type I interferons, exhibited strong antiviral activity against a broad spectrum of viral infections, both in an interferon-dependent and interferon-independent manner [2]. IRF2 was linked to the development of various cancers like colorectal, pancreatic, and hepatocellular carcinoma (HCC), by controlling the transcription of genes such as *TP53*, *CXCL3*, *Bcl-2*, and *Bax* [3,4,5]. IRF3, IRF5, and IRF7 were essential for the production of type I interferons (IFNs) upon pathogen recognition by receptors, impacting viral and bacterial infections, inflammatory responses and autoimmune diseases, cancer growth, and metastasis, and changes in the tumor microenvironment [6,7,8,9]. IRF4 and IRF8 contributed to tumor immunity by modulating the functions of T cells, B cells, and NK cells [10,11]. IRF6, with its unique helix-turn-helix DNA-binding motif, primarily governs processes in limb and stomatofacial development, with limited documentation on its role in regulating IFNs in higher vertebrates [12]. IRF9 regulated the expression of interferon-stimulated genes (ISGs), which served as markers for type I and III interferon activation [13,14]. IRF10 was only observed in fish and birds, while IRF11 seemed to be a fish-specific member of the IRF family and will not be discussed in this review [15].

Recent research has shown that IRFs play a crucial role in the advancement and exacerbation of various liver diseases, such as hepatic ischemia-reperfusion injury (IRI), alcohol-induced liver injury, Con A-induced liver injury, nonalcoholic fatty liver disease (NALFD), cirrhosis, and HCC. IRF1, a nuclear transcription factor, was involved in various liver diseases, particularly hepatic IRI and HCC. It played a crucial role in regulating gene expression during inflammation and was up-regulated in both cold and warm liver IRI. IRF1 exerted deleterious effects by inducing the expression of inflammatory mediators, but liver grafts deficient in IRF1 showed significantly reduced liver injury [16,17]. Specifically, in the early post-transplantation period, IRF1 overexpression in donor livers led to elevated caspase 3 expression, whereas knockdown of IRF1 reduced caspase 8 activity, resulting in a significant reduction in hepatocyte apoptosis and liver injury. In addition, IRF1 was involved in the hepatic IRI process through the regulation of various immune cell activities (NK and T cells), cytokines (IL-6, IL-12, IL-15, IL-23), and death ligands, as well as oxidative stress [18,19,20,21]. IRF1 also served as a key transcription factor that controlled gene expression in both innate and adaptive immunity, responding to type I (IFN-α/β) and type II (IFN-γ) interferons [22,23]. It was involved in IFN-γ-induced apoptosis of tumor cells by triggering downstream signals like inducible nitric oxide synthase (iNOS), p21, p53, p53-upregulated modulator of apoptosis (PUMA), and Fas-related death domain [24,25,26,27,28]. Yan, Y. et al. showed that IRF1 promotes the migration of CD8^+^ T cells and NK and NKT cells and stimulates IFN-γ secretion in NK and NKT cells, ultimately leading to tumor cell apoptosis via the CXCL10/CXCR3 pathway [29]. However, Wang, R. et al. found that gamma interferons can upregulate IRF1 expression in HCC cells, which in turn activates human endogenous retrovirus-H long terminal repeat-associating 2 (HHLA2) expression and promotes M2 polarization and macrophage chemotaxis, potentially facilitating immune evasion and HCC progression [30]. IRF1 may have a dual role in HCC, which requires further experimental verification. In addition to IRF1, other members of the IRF family have also been discovered to play significant roles in various liver diseases, demonstrating diverse functions in either promoting or inhibiting these diseases. Consequently, a comprehensive understanding of the role of IRFs in liver disease can offer valuable insights into disease mechanisms and potential therapeutic targets.

### Materials and Methods

A systematic search was conducted in the PubMed electronic database to gather relevant literature. Additionally, the reference lists of the primary studies included were reviewed to identify potentially eligible articles. Only articles published in English were considered, with no restrictions on publication year. The index terms encompassed various topics such as ‘interferon regulatory factor’, ‘IRF’, ‘ischemia reperfusion injury’, ‘hepatic ischemia reperfusion injury’, ‘liver injury’, ‘hepatic injury’, ‘NAFLD’, ‘nonalcoholic fatty liver disease’, ‘cirrhosis’, ‘liver fibrosis’, ‘HCC’, and ‘hepatocellular carcinoma’. A critical evaluation was performed on all studies included in this paper.

## 2. The Structure and Function of IRFs

The eleven members of the IRF family, IRF1 to IRF11, have been identified, with their primary roles centered around transcriptional regulation in the immune system and cell growth [31,32,33]. All IRFs share common domains, including an N-terminal helical DNA-binding domain (DBD) with five conserved tryptophan repeats, and a C-terminal IRF-associated domain (IAD) that facilitates protein interactions and modulates various transcriptional activities [31,34,35]. Each member of the IRF family plays a distinct role in various biological processes, including pathogen response, cytokine signaling, cell growth regulation, hematopoietic development, immune cell development, differentiation, and apoptosis [36]. In-depth study of the functions of IRFs is crucial for comprehending the mechanisms underlying disease occurrence and progression.

IRF1, a pleiotropic transcription factor, exhibited high responsiveness to various stimuli such as IFN, NF-κB, TBK-1, and IKK-ɛ, undergoing rapid and dynamic regulation upon infection [37,38,39,40]. Despite the short life cycle of IRF1 mRNA and protein, it consistently expressed a variety of host defense genes and effectively drove the expression of genes related to innate immunity [31,41,42,43]. Type I/II IFN stimulated hepatocytes to release IL-7, which in turn limited IRF1, enhanced the secretion of pro-inflammatory cytokines induced by lipopolysaccharide (LPS), and reduced macrophage tolerance to LPS [44]. Similar to IRF1, IRF2 competed for the same cis-elements and inhibited the function of IRF1, thereby reducing the production of IRF1-dependent pro-inflammatory genes such as IL-12, IFNβ, and iNOS, which ultimately helped in reducing hepatic IRI [45]. IRF3, a crucial component of the innate immune response, was responsible for detecting and reacting to foreign antigens, thereby helping to prevent viral infections [46]. Besides its role in gene transcription, IRF3 also initiated non-transcriptional and pro-apoptotic signaling pathways. Although the pro-apoptotic activity of IRF3 was beneficial in fighting viral infections, it could also lead to liver cell death and exacerbate detrimental immune responses in liver disease [47]. IRF4 was an oncogenic master transcription factor that induced cancer transcriptional programs by forming unique regulatory circuits that interacted with upstream pathways and binding partners [10]. The expression of IRF4 and PD-1 was positively correlated, and the overexpression of IRF4 was found to impede the proliferation and migration of HCC by suppressing the JAK2/STAT3 signaling pathway and epithelial-mesenchymal transition [48]. Furthermore, IRF4 served as a crucial transcriptional regulator of lipid processing in adipocytes and acted as a suppressor of inflammation in diet-induced obesity. Reduced IRF4 expression was associated with the exacerbation of NAFLD, leading to heightened levels of inflammation and insulin resistance [49]. IRF5 played a central role in inflammation and was recruited to promote inflammatory genes with the assistance of the NF-kB p65 sub-unit RelA, inducing pro-inflammatory cytokines such as IL-6, IL-12, IL-23, and TNF-a [50,51,52]. Highly expressed in monocytes, macrophages, B-cells, and dendritic cells, IRF5 was implicated in various inflammatory and autoimmune diseases [51,53,54,55]. Alzaid, F. et al. showed a significant increase in IRF5 expression in hepatic macrophages of individuals with non-alcoholic fatty liver disease or hepatitis C virus infection, contributing to liver fibrosis. Interestingly, mice lacking IRF5 in myeloid cells were found to be protected from stress-induced hepatic fibrosis, suggesting the crucial role of IRF5 in hepatocellular cell death and liver fibrosis in both mice and humans [56]. The tumor suppressor IRF6 was not implicated in IFN gene regulation and was notably decreased during epithelial-mesenchymal transition (EMT) in gastric and pancreatic ductal adenocarcinomas. This downregulation was controlled by the transcription factor ZEB1 [57,58]. In hepatocytes, IRF6 directly interacted with the promoter of the peroxisome proliferator-activated receptor γ (*PPARγ*) gene, leading to the inhibition of *PPARγ* transcription and its associated target genes involved in lipogenesis and fatty acid uptake [59]. Moreover, lower IRF6 expression was observed in colorectal cancer tissues and liver metastases compared to normal tissues. Mechanistically, IRF6 may enhance cisplatin-mediated cell proliferation, migration, invasion, and apoptosis sensitivity to chemotherapy by regulating E-calmodulin and Ki67 [60]. IRF7, initially recognized as a key transcription factor in the production of type I IFNs and regulation of the innate immune response, was situated on chromosome 11p15.5 in humans and chromosome 7 F5 in mice [7,61]. With a diverse array of functions, IRF7 was a pivotal component in the type I/III IFNs induced signaling pathway, contributing significantly to viral infections, autoimmune diseases, and the maintenance of homeostasis [7]. IRF8 played a crucial role in regulating immune cell differentiation and inducing innate immunity. It collaborated with IRF1, IRF2, IRF4, and other transcriptional regulators like TEL, PU.1, MIZ1, and E47 to modulate gene transcription by influencing the formation of DNA-binding compounds and controlling the expression of downstream target genes [62,63,64]. IRF8 knockout mice showed a reduction in inflammatory cell infiltration and cytokine release, leading to improved outcomes after liver IRI. Conversely, overexpression of IRF8 was linked to worsened liver damage and functional abnormalities [65]. Interestingly, in HCC cells, IRF8 overexpression demonstrated enhanced antitumor effects by potentially regulating tumor-associated macrophages and T cell levels in the tumor microenvironment [66]. IRF9 interacted with peroxisome proliferator-activated receptor α (PPARα) and activated PPARα target genes to attenuate inflammation, hepatic steatosis, and insulin resistance [67]. However, IRF9 also suppressed SIRT1 expression, resulting in increased p53 protein acetylation, ultimately exacerbating hepatic IRI [68]. These findings suggest that the role of IRFs may vary depending on the specific liver disease being studied.

## 3. IRFs and Liver Diseases

IRFs are a group of multifunctional transcription factors that target IFN promoters and interferon-stimulated response elements (ISREs) in ISGs, activating the expression of target genes. This indicates the essential role of IRFs in a range of biological processes including antiviral defense, innate immunity, adaptive immunomodulation, cell growth, apoptosis, and homeostasis maintenance [69]. Despite their known functions, the involvement of IRFs in the regulation of liver diseases has yet to be explored (Table 1).

### 3.1. IRFs and Liver Ischemia-Reperfusion Injury

IRI was a common complication of liver transplantation, partial hepatectomy, and hypovolemic shock. This phenomenon resulted in complex hepatocellular damage, early graft dysfunction, and an increased risk of acute and chronic rejection, ultimately resulting in poor prognosis and low patient survival rates [114,115]. Treatment for IRI was primarily supportive, as there were no specific drugs or methods available. Various strategies have been explored to address liver IRI, such as ischemic preconditioning, pharmacological and surgical interventions, and gene therapy [116]. Despite advancements in graft management targeting autophagy, oxidative stress, sterile inflammation, and apoptosis, the intricate pathological mechanisms of IRI remained inadequately understood.

#### 3.1.1. Autophagy and Oxidative Stress

IRF1 and IRF5 expression were found to be significantly increased in the liver following exposure to IRI [75,81]. IRF1 had been shown to have a detrimental impact on hepatic IRI by regulating the expression of various inflammatory mediators. Overexpression of IRF1 in donor livers led to elevated expression of caspase 3 during the early post-transplantation period. Conversely, knockdown of IRF1 resulted in reduced mRNA levels of death ligands and receptors in hepatocytes, as well as decreased caspase 8 activity, leading to a notable decrease in hepatocyte apoptosis and liver injury [16,17,20]. Yu, Y. et al. demonstrated that IRF1 played a critical role in promoting P62 expression by activating autophagy through P38 phosphorylation, leading to hepatocyte death [77]. Additionally, Yan, B. et al. showed that IRF1 overexpression induces autophagy, suppresses β-catenin expression, and worsens hepatic IRI [73]. Furthermore, IRF1 exacerbated hepatic IRI via JNK-mediated autophagy, resulting in increased Beclin1 levels. Conversely, silencing IRF1 reduces high mobility group box 1 (HMGB1) expression and release in the liver, decreases LC3II and Beclin1 levels, and mitigates hepatic IRI [72,75]. Reactive oxygen species (ROS) also played a crucial role in IRI of the liver, primarily originating from Kupfer cells and mitochondria during IRI [117]. Elevated hepatic ROS levels at the onset of IRI triggered the expression of IRF1, promoting the transcription of HMGB1. Acetylated HMGB1 then activated the downstream TLR-4/NF-κB signaling pathway, resulting in the release of inflammatory mediators like TNF-α and IL-1β, worsening hepatic IRI damage [70]. The inhibition of IRF1-mediated HMGB1 release and subsequent TLR activation or p38 MAPK signaling pathway inactivation was shown to prevent hepatic IRI [71,79]. In addition, IRI upregulated iNOS and promoted the transcriptional activity of IRF1 through HDAC2-mediated histone H3 acetylation, which led to cell death and tissue injury [74]. Moreover, IRF1 regulated the transcription of *Rab27a* (a guanosine triphosphatase) and extracellular vesicle secretion, leading to oxidized phospholipids activation in neutrophils and subsequent hepatic IRI [76]. Thus, autophagy and oxidative stress play an important role in hepatic IRI, but it has not been clearly elucidated, and more studies are still needed.

#### 3.1.2. Inflammatory Cytokines

IRI first triggers pro-inflammatory signaling cascades like TNF-α, IL-6, IFN, and NF-kB. As liver cells perish, cytokines and chemokines are produced, leading to a chemical storm that attracts neutrophils and other immune cells to the liver. This recruitment further stimulates the release of CXCL1, CXCL2, and complement, exacerbating the IRI [118]. Moreover, IRI activates the innate immune system, which drives the overall development of inflammatory hepatocellular injury. Further exploration of the roles played by immune cells and inflammatory cytokines in IRI is crucial for us to design safe and effective therapeutic strategies to ameliorate IRI in patients [119]. In recent years, it has been found that multiple IRF family members can promote the progression of IRI by regulating interleukin production and immune cell infiltration. Yokota, S. et al. discovered that knocking down IRF1 led to a decrease in the population of NK, NKT, and CD8^+^ T cells in the liver. Additionally, they observed that IRF1 increases cytotoxic effects and systemic inflammatory responses, worsening hepatic injury by controlling the expression of *IL-15* and *IL-15Rα* mRNA in mouse hepatocytes and hepatic dendritic cells [78]. Another study by Nakano, R. et al. demonstrated that overexpression of IRF1 in ApoE^-/-^ mice exacerbated hepatic IRI injury by activating hepatic NK and T cells through IL-15 production [18]. Additionally, early induction of IL-23 in hepatic IRI activated the IFN-γ/IRF1 pathway, leading to increased apoptosis and necrosis [21]. Type I IFNs were found to up-regulate hepatic IRF1 expression, which in turn regulated apoptosis and induced hepatic injury after IRI. Deprivation of plasmacytoid dendritic cells (pDC) in mice after IRI resulted in milder hepatic injury, reduced levels of hepatic IL-6, TNF-α, and apoptosis, and impaired expression of IRF1 and pro-apoptotic molecules such as Fas ligand, Fas, and death receptor 5 [19]. Therefore, IRF1 played a crucial role in hepatic IRI by regulating immune cells and their cytokines, making it a potential target for mitigating IRI. In addition, endogenous IRF2 was typically expressed in the liver and can be slightly upregulated by liver IRI. Overexpression of IRF2 was shown to protect against hepatic IRI and also restricted the production of IRF1-dependent proinflammatory factors such as IL-12, IFNβ [45]. Loi, P. et al. found that IRF3-deficient mice exhibited heightened hepatic necrosis and increased neutrophil infiltration due to elevated expression of *IL-12*/*IL-23p40*, *IL-23p19*, and *IL-17A* mRNA, along with reduced expression of *IL-27p28* mRNA [80]. Wang, P.-X. et al. also found that IRF9 decreased the expression of SIRT1 and increased the level of acetyl p53, leading to a significant increase in immune cell infiltration, inflammatory cytokine levels and hepatocyte apoptosis [68]. Therefore, an in-depth study of the role of IRFs in IRI can help us better understand the mechanisms of disease development and develop feasible therapeutic measures.

### 3.2. IRFs and Alcohol-Induced Liver Injury/Alcoholic Liver Disease

Alcoholic-induced liver injury (ALI) is typically identified by disrupted liver function, inflammatory cell accumulation, and oxidative stress. Clinically, alcoholic liver disease (ALD) is the predominant form of ALI. The abuse of alcohol leads to liver injury through various mechanisms, such as oxidative and non-oxidative ethanol metabolism, the production of oxidative stress, damage and dysfunction of mitochondria and lysosomes, endoplasmic reticulum stress, inflammation, cytokine release, and the triggering of cell death [120]. Approximately 90% of individuals with alcoholism develop hepatic steatosis, with 35% of those individuals progressing to alcoholic hepatitis. Unfortunately, 40% of patients diagnosed with severe alcoholic hepatitis do not survive beyond 6 months despite receiving treatment [121]. Regrettably, corticosteroids continue to be the conventional treatment for severe alcoholic hepatitis, showing no advancements in the past forty years [121,122]. It is imperative to enhance our comprehension of the pathogenic mechanisms underlying ALD, particularly the liver injury it induces, to formulate more efficient treatment approaches. Luther, J. et al. discovered that mice fed alcohol showed heightened hepatic expression of the cGAS-IRF3 pathway. And they observed that the downregulation of connexin 32 (the predominant hepatic gap junction) led to a decrease in IRF3 expression, ultimately leading to a decrease in liver injury as evidenced by a notable reduction in ALT/AST levels [89]. Furthermore, IRF1-mediated caspase 1 inflammatory vesicles and NOX2-dependent ROS pathways were found to exacerbate ALI and steatosis [87]. In addition, excessive alcohol consumption led to dysbiosis of the gut microbiota, compromising the integrity of the intestinal epithelium and facilitating the transport of gut microbial products (e.g., LPS) to the liver, which was recognized by Toll-like receptor-4 (TLR4), ultimately leading to liver injury and subsequent ALD [88,123]. Liang, S. et al. demonstrated that chronic ethanol intake and LPS injection resulted in elevated serum ALT and IL-1 levels, along with enhanced hepatic CCL5 and CXCL10 expression. In normal conditions, macrophages could facilitate IRF1 degradation through autophagy, eliminate damaged mitochondria, and restrict macrophage activation and inflammation. However, following p62 silencing or myeloid cell-specific autophagy-related 7 knockout, IRF1 accumulation occurred in autophagy-deficient macrophages and translocated into the nucleus, leading to increased expression of CCL5 and CXCL10 [88]. Petrasek, J. et al. showed that systemic IRF3 knockout mice were protective against ALI, steatosis, and inflammation, but knockout of IRF3 only in parenchymal cells exacerbated ALI. Hepatic parenchymal cells were further found to be a major source of type I IFNs, whose action was dependent on TLR4/IRF3. Meanwhile, type I IFNs potentiated LPS-induced IL-10 and inhibited inflammatory cytokine production in mouse macrophages and human leukocytes. Thus, IRF3 activation in liver parenchymal cells and the resulting type I IFNs are protective against ALD by modulating the inflammatory function of macrophages [124]. However, Sanz-Garcia, C. et al. found that Gao-binge ethanol exposure activated IRF3 signaling and led to liver injury. IRF3 was further found to have a non-transcriptional function and could be induced to bind to Bax in mitochondria and activate caspases 3 and 9, which in turn activated the apoptotic pathway and limited NF-κB activity [125]. Therefore, IRF3 could regulate the innate immune environment of the liver and alleviate ALI/ALD by increasing apoptosis of immune cells.

### 3.3. IRFs and Con A-Induced Liver Injury

Conjugin A (Con A), a plant lectin isolated from the miller bean (Canavalia ensiformis), was recognized for its role as a T-lymphocyte activator in the immune response to allograft rejection, viral infections or autoimmune disorders in mammals [126]. Con A stimulated the release of a variety of cytokines from immune cells (such as TNF-α, IFN-γ, GM-CSF, IL-2, IL-1β), induced oxidative stress, activated multiple signaling pathways (NF-κB, MAPK, PI3K/PDK1/mTOR, STAT3, and STAT5), and altered the Bax/Bcl-2 ratio, ultimately resulting in severe liver inflammation and hepatocyte apoptosis/necrosis [83,127,128,129,130]. However, the precise cellular mechanisms underlying liver dysfunction induced by Con A activation remained incompletely understood. Recent studies have revealed the involvement of IRFs in Con A-induced liver injury. Binding of Con A to the mannose 6-phosphate receptor of HSCs induced JAK2/STAT-1 phosphorylation and promoted IRF1 transcription, which in turn inhibited superoxide dismutase (SOD) expression and promoted JNK and caspase 3 activation, leading to oxidative stress and apoptosis in hepatocytes [82]. In a study involving mice injected with Con A, elevated levels of IL-28A were observed. Deficiency in IL-28A was found to limit M1 macrophage polarization by modulating a signaling pathway that inhibits IRF5, consequently reducing the release of pro-inflammatory cytokines such as TNF-α, IL-12, IL-6, and IL-1β from M1 macrophages [83]. Corilagin was shown to effectively inhibit the release of pro-inflammatory cytokines in M1 macrophages by restraining the activation of the IRF5 signaling pathway, providing protection against Con A-induced immune-mediated liver injury. This treatment also resulted in reduced expression of M1 macrophage-associated pro-inflammatory cytokines and genes, including IL-6, IL-12, and iNOS [84]. Additionally, in Con A-induced hepatitis, the absence of liver X receptor α (LXRα) resulted in an increase in MDSCs in the liver, which in turn attenuated liver injury. Mechanistically, MDSCs from LXRα^-/-^ mice exhibited significantly lower expression of IRF8, facilitating the expansion of MDSCs and effectively reducing immune injury in the liver [85].

### 3.4. IRFs and Post-Transplantation/Other Modes of Liver Injury

Studies have shown that increased IRF1 and IRF4 expression in patients undergoing acute rejection post-liver transplantation contributes to liver inflammation and injury [91,131,132]. Moreover, treatment with tacrolimus (TCA) suppresses IRF4 expression, thereby alleviating acute rejection after liver transplantation. This effect may be mediated through the TAC-NFAT-IRF4 and BATF/JUN/IRF4 complex-IL-21 axes, with the latter inhibiting IL-12-producing Tfh cells and consequently reducing liver injury [91,92]. Zhao, W. et al. also demonstrated that silencing IRF4 resulted in decreased levels of inflammatory factors such as TNF-α, IL-6, and IFN-γ and induced anti-inflammatory IL-10 levels, thereby attenuating acute liver transplant rejection in mice [133]. In adult patients with acute hepatitis A caused by HAV infection, severe liver injury was observed with elevated levels of chemokines such as CXCL10, CCL4, and CCL5. However, inhibiting IRF3 expression was found to decrease CXCL10 production, thus alleviating liver injury [90]. Similarly, in patients diagnosed with primary sclerosing cholangitis and primary biliary cirrhosis, bile acids promoted IRF3 phosphorylation, resulting in elevated expression of the target gene ZBP1. This ultimately exacerbated hepatic injury and fibrosis through interaction with RIP1, RIP3, and NLRP3 [86]. Additionally, in an LPS-induced liver injury model in mice, IRF3 expression was increased, a transcription factor was linked to systemic inflammation, while B-HA was shown to attenuate LPS-stimulated inflammatory responses by inhibiting the activation of the TLR4 signaling pathway through the phosphorylation of IRF3 [134,135]. In summary, IRFs play an important role in liver injury caused by a variety of etiological factors, and in-depth study of the molecular mechanisms of IRFs can help to understand the occurrence of liver injury and develop appropriate therapeutic strategies.

### 3.5. IRFs and Nonalcoholic Fatty Liver Disease

Inflammation has significant implications for metabolism, particularly in the context of obesity and NAFLD. Studies on NAFLD mice induced with a high-fat, high-fructose diet revealed the progression of simple steatosis, steatohepatitis, and hepatic fibrosis over 4, 8, and 16 weeks, respectively. The expression of IRF3 and IRF7 showed an increase at week 4, peaked at week 8, and returned to basal levels by week 16 [136]. However, research on liver tissues from 11 patients with NAFLD and 11 controls did not show a significant difference in IRF3 expression [137]. In mice on an HFD diet, systemic knockdown of IRF3 prevented steatosis and glycemic abnormalities, while hepatocyte-specific knockdown of IRF3 only impacted glycemic abnormalities. Mechanistically, IRF3-mediated Ppp2r1b induced an increase in PP2A activity, leading to AMPKα and AKT dephosphorylation [93]. Knockdown of IRF3 in mouse livers with HFD and FFA-induced L-O2 cells resulted in reduced hepatic inflammation and apoptosis, potentially through the regulation of the NF-κB signaling pathway, inflammatory cytokines, and apoptotic signaling [94]. Moreover, obese NAFLD patients show heightened hepatic IRF3 activation, which can be reversed with bariatric surgical treatment [93]. In a NASH mouse model, skeletal muscle-specific IRF4 knockout mice displayed improvements in hepatic steatosis, inflammation, and fibrosis without affecting body weight. IRF4 plays a role in transcriptionally regulating FSTL1, establishing a connection between muscle and liver [93]. In the HFD-induced NAFLD model, hepatic IRF6 was inhibited by promoter hypermethylation, and hepatocyte-specific transgenic mice overexpressing IRF6 exhibited attenuated steatosis and metabolic disease. Mechanistically, hepatocyte IRF6 bound directly to the promoter of the *PPARγ* gene and subsequently stopped transcription of *PPARγ* and its target genes (regulating adipogenesis and fatty acid uptake), resulting in amelioration of NAFLD progression [59].

### 3.6. IRFs and Liver Fibrosis

Liver fibrosis is a precursor to cirrhosis and results from extracellular mesenchymal protein deposition, activated hepatic stellate cells (HSC) are essential for the development of liver fibrosis, and liver inflammation triggered by activation of liver resident macrophages and massive leukocyte aggregation are also associated with liver fibrosis-related acute and chronic liver injury. A study revealed a lower frequency of the AG genotype of the IRF3(-925A/G) gene in cirrhotic patients, suggesting a potential protective effect against HCV infection [138]. Yu, J. et al. discovered that Gαs-coupled GPCR signaling increased IRF3 phosphorylation through cAMP-mediated PKA activation, resulting in elevated IL-33 expression, ultimately promoting HSC activation, and subsequently contributing to hepatic fibrosis progression [99]. Iracheta-Vellve, A. et al. demonstrated that in CCL4-treated hepatocytes, endoplasmic reticulum (ER) stress via STING triggered TBK1 phosphorylation, followed by IRF3 phosphorylation, which then interacted with BAX in mitochondria through its BH3-only structural domain, leading to pro-apoptotic caspase 3 activation and hepatocyte apoptosis, ultimately contributing to liver fibrosis [98]. However, Wu, Q. et al. found that STING increased IRF3 phosphorylation via TBK1, subsequently inhibiting CDK4/6-mediated RB hyperphosphorylation and inactivating E2F transcription factors, thereby inducing senescence in HSC cells. Interestingly, total knockdown or conditional deletion of IRF3 in HSC exacerbated liver fibrosis, indicating a dual role for IRF3 in this process that required further investigation [100]. Moreover, INF-γ was found to significantly upregulate indoleamine 2,3-dioxygenase (IDO) expression through STAT1 activation, leading to tryptophan depletion and subsequent G1 cell cycle arrest. Upon release from IFN-γ-induced G1 cell cycle arrest by 1-MT treatment, HSC apoptosis was significantly increased due to enhanced IRF1 expression [97]. Similarly, GRh2 attenuates hepatic inflammation and fibrosis by up-regulating IRF1 expression, which inhibits SLC7A11 and promotes HSC iron death and inactivation [96]. In human hepatocyte macrophages with liver fibrosis from NAFLD or HCV infection, elevated IRF5 expression triggered the release of inflammatory cytokines and death effectors, leading to hepatocyte caspase-dependent apoptosis. Conversely, IRF5-deficient macrophages under hepatocyte stress exhibited immunosuppressive polarization, secreting IL-10 and TGFβ to support BCL2 family member-mediated anti-apoptotic signaling in hepatocytes during metabolic or toxic stress-induced liver fibrosis [56].

### 3.7. IRFs and Hepatocellular Carcinoma

HCC posed a significant global health challenge due to its high fatality rate [139,140]. Despite undergoing combination therapies such as radiochemotherapy and immunotherapy, the 5-year survival rate for HCC remained notably low [141]. IRF1 and IRF2 were essential in regulating interferon activity, where the absence of IRF1 and the increase in IRF2 expression had been associated with aggressive traits in different types of cancer. IRF1, acting as a tumor suppressor gene, enhanced the migration of CD8^+^ T-cells, NK cells, and NKT cells, while also stimulating IFN-γ secretion in NK and NKT cells. This activation led to apoptosis in tumor cells through the CXCL10/CXCR3 paracrine axis [29]. Conversely, the downregulation of IRF2 significantly reduced invasive capacity, correlating with the decreased expression of STAT3, p-STAT3 and MMP9 [142]. However, increased *IRF1* mRNA expression was observed in patients with highly differentiated or early HCC. In vitro studies demonstrated that IFN-γ induced PD-L1 mRNA and protein expression by enhancing IRF1 levels in both mouse and human HCC cells, whereas IRF2 overexpression down-regulated IFN-γ-induced PD-L1 promoter activity and protein abundance. Additionally, IRF1 was found to antagonize IRF2 binding to the IRE promoter element in PD-L1, leading to the upregulation of PD-L1 in the tumor microenvironment [143]. Furthermore, IRF1 was found to post-transcriptionally suppress and induce apoptosis in HCC cells by facilitating the interaction between miR-195 and the 3′UTR of checkpoint kinase 1 (CHK1). However, IRF1 also enhanced PD-L1 expression by promoting STAT3 phosphorylation [106]. Therefore, elevated IRF-1 expression not only triggered apoptosis in HCC cells but also boosted PD-L1 levels, shedding light on the regulation of the PD-L1/PD-1 pathway in HCC therapy. In both human and mouse HCC cells, IL-33 overexpression was shown to inhibit proliferation and decrease PD-L1 levels at the transcriptional level by enhancing the ubiquitin-dependent degradation of IRF1. This process was disrupted by E3 ligase RanBP2-mediated SUMOylation of IL-33 at Lys54 [102]. Additionally, treatment with cisplatin and CHK1 inhibitors led to the upregulation of major histocompatibility complex (MHC) class I associated chains A (MICA) expression through IRF1-mediated transcriptional effects, resulting in increased infiltration of NK cells and CD8^+^ T cells in HCC tissues [104]. FOXO1 functioned as a tumor suppressor by promoting macrophage infiltration and antitumor polarization via positive regulation of the IRF1/NO pathway. Polarized macrophages further inhibited HCC proliferation and migration by suppressing IL-6/STAT3 activation [105]. NR4A1 was markedly upregulated in tumor-infiltrating NK cells, which reduced the efficacy of anti-PD-1 therapy through modulation of the IFN-γ/p-STAT1/IRF1 signaling pathway, leading to dysfunctional tumor-infiltrating NK cells [103]. Moreover, high expression levels of miR-23a, miR-31, and miR-301a in HCC enhanced its progression by inhibiting IRF1 expression [107,108,109]. Conversely, miR-345 showed low expression in HCC, counteracting the inhibitory impact of IRF1 through reversible trans-overexpression, thereby influencing the epithelial-mesenchymal transition process and tumor metastasis [101]. Phosphatase and tensin homolog (PTEN) in HBV-associated HCC regulated the nuclear localization of IRF3 by dephosphorylating IRF3 at ser97, leading to the inhibition of the PI3K/AKT pathway and the reduction of oncogenic effects [110]. IRF4 overexpression suppressed the proliferation and migration capabilities of HCC cells by inhibiting the JAK2/STAT3 signaling pathway and epithelial-mesenchymal transition [48]. IRF5, identified as a tumor suppressor, exhibits down-regulated mRNA and protein expression levels in HCV-infected human hepatocytes and cells with autonomous replication of HCV RNA. Conversely, restoration of IRF5 expression hampered HCV protein translation and RNA replication [111]. However, Fang, Y. et al. observed an upregulation of IRF5 expression in HCC, facilitating an oncogenic effect by enhancing glycolysis through upregulation of lactate dehydrogenase A (LDHA) expression [112]. In addition, miR-424-3p reduced the interferon pathway by attenuating the transcriptional activation of STAT1/2 and IRF9 genes by SRF, which in turn enhanced matrix metalloproteinases (MMPs)-mediated ECM remodeling [113]. Therefore, IRF1, IRF3, IRF5, and IRF9 are all involved in the development of HCC, and further research into the molecular mechanisms involving IRFs in carcinogenesis could facilitate the discovery of new targeted therapies.

## 4. Conclusions and Future Perspectives

In summary, IRF1, IRF2, IRF3, IRF5, IRF8, and IRF9 are involved in hepatic IRI; IRF1, IRF5, and IRF8 are involved in Con A-induced liver injury; IRF3 is involved in cholestasis-induced liver injury and HAV-induced liver injury; IRF4 is involved in liver transplantation-induced liver injury; IRF1 and IRF3 are involved in alcohol-induced liver injury; IRF3, IRF4, and IRF6 are involved in the development of NAFLD; IRF1, IRF3, and IRF5 are involved in the development of liver fibrosis; and IRF1, IRF3, IRF4, IRF5, IRF8, and IRF9 are involved in the development of HCC. Almost all members of the IRF family play a role in different liver diseases, especially IRF1, which has been the most studied. IRF7 is less well-studied in liver diseases, and its expression is only increased in certain disease processes and usually accompanied by IRF3. Although the family of IRFs plays a very important role in liver diseases, there are no targeted drugs against IRFs to treat the disease process. In addition, the same IRFs may play different functions in different liver diseases, promoting or inhibiting the disease. Therefore, further clarification of the molecular mechanisms of IRFs in liver diseases is needed in the future to guide clinical practices and the development of corresponding targeted drugs.

## Figures and Tables

**Table 1:** The function and mechanism of IRFs in liver diseases.

First Author	Publication Year	Disease	Model	Stimulation	State of IRFs	Involving Molecules	Signal Pathway	Effects
Nakano, R. [18]	2024	Hepatic Ischemia-Reperfusion Injury	mice	ApoE^-/-^	upregulation of IRF1	IL-15	-	Atherosclerosis can mirror intrahepatic immunity, particularly activating liver NK and T cells through IL-15 production, thereby exacerbating hepatic damage. The upregulation of IL-15 expression is associated with IRF1 overexpression.
Li, K. [70]	2023	Hepatic Ischemia-Reperfusion Injury	Sprague-Dawley rat	Chlorogenic acid (CGA)	deregulation of IRF1	HMGB1	-	CGA pretreatment significantly decreased the levels of reactive oxygen species following HIRI, inhibited HMGB1 release by decreasing IRF1 expression, inhibited the expression of HMGB1, TLR-4, MyD88, P-IκB-α, NF-κB P65, and P-P65, and promoted IκB-α degradation. Thus, CGA appears to inhibit oxidative stress and inflammatory responses during HIRI.
Luo, J. [71]	2022	Hepatic Ischemia-Reperfusion Injury	mice	PIAS1	deregulation of IRF1	p38	PIAS1/NFATc1/HDAC1/IRF1/p38 MAPK	PIAS1 inactivated p38 MAPK signaling by inhibiting HDAC1-mediated IRF1 through NFATc1 SUMOylation, thereby repressing the inflammatory response and apoptosis of hepatocytes in vitro, and alleviating liver injury in vivo.
Li, S. [72]	2021	Hepatic Ischemia-Reperfusion Injury	mice	-	upregulation of IRF1	JNK, Beclin1	IRF1/JNK	IRF1 is associated with JNK pathway activation followed by increases in Beclin1 protein levels. This JNK-induced autophagic cell death then leads to cell failure and plays an important role in liver function damage.
Yan, B. [73]	2020	Hepatic Ischemia-Reperfusion Injury	mice	-	upregulation of IRF1	β-catenin	-	IRF1-induced autophagy aggravates hepatic IR injury in part by inhibiting β-catenin.
Du, Q. [74]	2020	Hepatic Ischemia-Reperfusion Injury	mice	iNOS/NO	upregulation of IRF1	iNOS, PUMA, p21	-	iNOS/NO-induced HDAC2 mediated histone H3 deacetylation and promoted IRF1 transcriptional activity.
Klune, J.R. [21]	2018	Hepatic Ischemia-Reperfusion Injury	mice	IL-23	upregulation of IRF1	-	-	The overexpression of IL-23 in vivo through the use of an adenovirus vector also led to the expression of IL-17, CXCL2, IFN-γ, and IRF1. The increased expression of IL-23 also led to increased apoptosis in the liver. IL-23 is induced early by I/R in the liver. Its signaling leads to the activation of the IL-17/CXCL2 and IFN-γ/IRF1 pathways, resulting in increased apoptosis and necrosis.
Cui, Z. [75]	2018	Hepatic Ischemia-Reperfusion Injury	mice	-	upregulation of IRF1	HMGB1	-	The levels of hepatic IRF1 mRNA and protein were significantly increased in livers after exposure to IRI, as well as an IRI-induced increase in *HMGB1* mRNA and release of HMGB1 in liver tissue. IRF1 activates autophagy to aggravate hepatic IRI by increasing HMGB1 release.
Yang, M.Q. [76]	2018	Hepatic Ischemia-Reperfusion Injury	mice	-	upregulation of IRF1	Rab27a, extracellular vesicle, OxPL	-	IRF1 regulates Rab27a transcription and EV secretion, leading to OxPL activation of neutrophils and subsequent hepatic IR injury.
Yu, Y. [77]	2017	Hepatic Ischemia-Reperfusion Injury	mice	-	upregulation of IRF1	P38	P38/P62	IRF1 functioned as a trigger to activate autophagy via P38 activation and P62 was required for this P38-mediated autophagy. IRF1 appears to exert a pivotal role in hepatic IRI, by predisposing hepatocytes to activate an autophagic pathway. Such an effect promotes autophagic cell death through the P38/P62 pathway.
Yokota, S. [78]	2015	Hepatic Ischemia-Reperfusion Injury	mice	-	upregulation of IRF1	IL-15/IL-15Rα	-	IRF1 promotes liver transplantation (LTx) I/R injury via hepatocyte IL-15/IL-15Rα production which suggests that targeting IRF-1 and IL-15/IL-15Rα may be effective in reducing I/R injury associated with LTx.
Castellaneta, A. [19]	2014	Hepatic Ischemia-Reperfusion Injury	mice	IFN-α	upregulation of IRF1	Fas ligand, its receptor (Fas) and death receptor 5	-	IFN-α derived from liver pDC plays a key role in the pathogenesis of liver I/R injury by enhancing apoptosis as a consequence of the induction of hepatocyte IRF1 expression.
Cho, H.I. [79]	2013	Hepatic Ischemia-Reperfusion Injury	mice	anethole	deregulation of IRF1	HMGB1/TLR	-	Anethole protects against hepatic I/R injury by the suppression of IRF1-mediated HMGB1 release and subsequent TLR activation.
Ueki, S. [17]	2010	Hepatic Ischemia-Reperfusion Injury	mice	-	deregulation of IRF1	caspase-8, IFN-γ	-	IRF1 deficiency in liver grafts, but not in recipients, resulted in a significant reduction in hepatocyte apoptosis and liver injury, as well as improved survival. Deficiency of IRF1 signaling in grafts resulted in significantly reduced messenger RNA (mRNA) levels for death ligands and death receptors in hepatocytes, as well as decreased caspase-8 activities, indicating that IRF1 mediates death ligand-induced hepatocyte death.
Kim, K.H. [20]	2009	Hepatic Ischemia-Reperfusion Injury	Sprague-Dawley rat	-	upregulation of IRF1	IFN-β, IFN-γ, IL-12, caspase-3	-	Rats pretreated with AdIRF-1 before transplantation had elevated alanine aminotransferase levels and increased expression of IFN-β, IFN-γ, IL-12, and iNOS in the short-term period (3 h) when compared with donor livers pretreated with Adnull.
Klune, J.R. [45]	2012	Hepatic Ischemia-Reperfusion Injury	mice	-	upregulation of IRF1	IL-12, IFN-β, iNOS	-	IRF2 overexpression limits the production of IRF1-dependent proinflammatory genes, such as IL-12, IFNβ, and iNOS, even in the presence of IRF1 induction. Additionally, isograft liver transplantation with IRF2 heterozygote knockout (IRF2^+/-^) donor grafts that have reduced endogenous IRF2 levels results in worse injury following cold I/R during murine orthotopic liver transplantation.
Loi, P. [80]	2013	Hepatic Ischemia-Reperfusion Injury	mice	-	deregulation of IRF3	IL-27p35, IL-27p28	IRF3/TLR4/IL-23/IL-17	Quantification of cytokine gene expression revealed an increased liver expression of *IL-12*/*IL-23p40*, *IL-23p19* mRNA, and *IL-17A* mRNA in IRF3-deficient versus wildtype mice, whereas *IL-27p28* mRNA expression was diminished in the absence of IRF3. IRF3-dependent events downstream of TLR4 control the IL-23/IL-17 axis in the liver and this regulatory role of IRF3 is relevant to liver ischemia-reperfusion injury.
Nasiri, M. [81]	2019	Hepatic Ischemia-Reperfusion Injury	mice	N-acetylcysteine	deregulation of IRF5	-	-	Pretreatment with N-acetylcysteine significantly decreased the mRNA levels of *TLR4/IRF5* and its downstream cytokines 3 h after reperfusion and subsequently improved the previously mentioned hepatic damages 168 h after reperfusion.
Shi, G. [65]	2021	Hepatic Ischemia-Reperfusion Injury	mice	-	upregulation of IRF8	CCXCL1, CXCL9, CCRL2	-	IRF8 is up-regulated in the early hypnotic stage of IR. Upregulated IRF8 activates NF-κВ signaling pathway and promotes the release of autophagy-dependent or -independent chemokines, which in turn recruit massive neutrophils into the damaged liver sites, exacerbating hepatic I/R inflammatory injury.
Wang, P.X. [68]	2015	Hepatic Ischemia-Reperfusion Injury	mice	-	upregulation of IRF9	SIRT1, p53	-	A deficiency in IRF9 markedly reduced the necrotic area, serum ALT, AST, immune cell infiltration, inflammatory cytokine levels, and hepatocyte apoptosis after liver I/R. IRF9 has a novel function of inducing hepatocyte apoptosis after I/R injury by decreasing SIRT1 expression and increasing acetyl-p53 levels.
Rani, R. [82]	2018	Con A-Induced Hepatic Injury	mice	Con A	upregulation of IRF1	SOD, JNK, caspase 3	-	Con A binding to the mannose 6-phosphate receptor (Man-R) on HSCs induces JAK2 phosphorylation, which then phosphorylates STAT-1. The activated STAT1 translocates into the nucleus and induces IRF1 transcription. IRF1 produced this way stimulates IFNβ transcription. IFNβ protein released by HSCs binds to the IFNαβ receptor on hepatocytes and instigates JAK2/STAT1 activation, followed by IRF1 synthesis. IRF1 (through a currently unidentified mechanism) inhibits SOD expression leading to oxidative stress. Oxidative stress and IFNαβ stimulate JNK and caspase 3 activation, and cause apoptosis of hepatocytes.
Zhang, J. [83]	2024	Con A-Induced Hepatic Injury	mice	IL-28A deletion	deregulation of IRF5	IL-1β, IL-6, IL-12, TNF-α	-	IL-28A deletion plays an important protective role in the Con A-induced acute liver injury model and IL-28A deficiency inhibits the activation of M1 macrophages by inhibiting the NF-κB, MAPK, and IRF signaling pathways.
Yan, F. [84]	2022	Con A-Induced Hepatic Injury	mice	Corilagin	deregulation of IRF5	IL-6, IL-12, TNF-a, iNOS	-	Corilagin protects mice from Con A-induced immune-mediated hepatic injury by limiting M1 macrophage activation via the MAPK, NF-κB, and IRF signaling pathways, suggesting corilagin as a possible treatment choice for immune-mediated hepatic injury.
Li, B. [85]	2021	Con A-Induced Hepatic Injury	mice	LXRa^-/-^	deregulation of IRF8	-	-	Abrogation of LXRα facilitated the expansion of MDSCs by downregulating IRF8, thereby ameliorating hepatic immune injury profoundly.
Zhuang, Y. [86]	2024	Cholestatic-Induced Hepatic Injury	mice	Bile acid	phosphorylation of IRF3	ZBP1	IRF3-ZBP1	IRF3 knockout (IRF3^-/-^) mice showed significantly attenuated liver and kidney damage and fibrosis compared to wide-type mice after bile duct ligation. ZBP1 interacted with RIP1, RIP3, and NLRP3, thereby revealing its potential role in the regulation of cell-death and inflammation pathways. Bile acid-induced p-IRF3 and the IRF3-ZBP1 axis play a central role in the pathogenesis of cholestatic liver and kidney injury.
Li, H. [87]	2024	Alcohol-Induced Hepatic Injury	mice	GRPR	upregulation of IRF1	Caspase-1, NOX2	-	The pro-inflammatory and oxidative stress roles of GRPR might be dependent on IRF1-mediated the Caspase-1 inflammasome and the NOX2-dependent reactive oxygen species pathway, respectively.
Liang, S. [88]	2019	Alcohol-Induced Hepatic Injury	mice	p62 silencing or ATG7 deletion	upregulation of IRF1	CCL5, CXCL10	-	Macrophage autophagy protects against ALD by promoting IRF1 degradation and removal of damaged mitochondria, limiting macrophage activation and inflammation. Upon p62 silencing or ATG7 deletion, IRF1 starts to accumulate in autophagy-deficient macrophages and translocates into the nucleus, where it induces CCL5 and CXCL10 expression.
Luther, J. [89]	2020	Alcohol-Induced Hepatic Injury	mice	Cx32 deletion	deregulation of IRF3	IFNβ, IFIT2, IFIT3	-	Disruption of Cx32 in ALD impaired IRF3-stimulated gene expression, resulting in decreased hepatic injury despite an increase in hepatic steatosis.
Sung, P.S. [90]	2017	Hepatitis A-Induced Hepatic Injury	HAV-infected cells	-	deregulation of IRF3	CXCL10	-	CXCL10 production was reduced by silencing the expression of RIG-I-like receptor signal molecules, such as mitochondrial antiviral signaling proteins and IRF3, in HAV-infected cells.
Tang, T. [91]	2021	Liver Transplantation-Induced Hepatic Injury	rats	Tacrolimus (TAC)	deregulation of IRF4	IL-21	BATF/JUN/IRF4 complex-IL-21	TAC inhibited the expression of the BATF/JUN/IRF4 complex and interacted with the promoter of BATF and IRF4. The BATF/JUN/IRF4 complex participated in the inhibition of IL-21-producing Tfh cells after treatment with TAC.
Tang, T. [92]	2015	Liver Transplantation-Induced Hepatic Injury	rats	TAC	deregulation of IRF4	-	TAC-NFAT-IRF4	TAC treatment prolonged the survival of liver allografts in recipients, significantly attenuating hepatic tissue injury and improving liver function. IRF4 expression in grafts was downregulated after TAC treatment.
Patel, S.J. [93]	2022	Nonalcoholic Fatty Liver Disease	mice	HFD	upregulation of IRF3	Ppp2r1b	IRF3-PPP2R1B	Global ablation of IRF3 in mice on a high-fat diet protects against both steatosis and dysglycemia, whereas hepatocyte-specific loss of IRF3 affects only dysglycemia. Integration of the IRF3-dependent transcriptome and cistrome in mouse hepatocytes identifies Ppp2r1b as a direct IRF3 target responsible for mediating its metabolic actions on glucose homeostasis. IRF3-mediated induction of Ppp2r1b amplified PP2A activity, with subsequent dephosphorylation of AMPKα and AKT.
Qiao, J.T. [94]	2018	Nonalcoholic Fatty Liver Disease	L-O2 cell	Knocking down STING	deregulation of IRF3	p-p65/p65, TNF-α, IL-6, and IL-1β	-	STING and IRF3 were upregulated in livers of HFD-fed mice and in FFA-induced L-O2 cells. Knocking down either STING or IRF3 led to a significant reduction in FFA-induced hepatic inflammation and apoptosis, as evidenced by the modulation of the NF-κB signaling pathway, inflammatory cytokines, and apoptotic signaling
Guo, S. [95]	2023	Nonalcoholic Fatty Liver Disease	IRF4 knockout (F4MKO) mice	-	deregulation of IRF4	FSTL1	IRF4-FSTL1-DIP2A/CD14	Skeletal muscle-specific IRF4 knockout (F4MKO) mice exhibited ameliorated hepatic steatosis, inflammation, and fibrosis, without changes in body weight, when put on a NASH diet. Dual luciferase assays showed that IRF4 can transcriptionally regulate FSTL1. Further, inducing FSTL1 expression in the muscles of F4MKO mice is sufficient to restore liver pathology.
Tong, J. [59]	2019	Nonalcoholic Fatty Liver Disease	mice	HFD	deregulation of IRF6	PPARγ	-	IRF6 is downregulated by the promoter, hypermethylation, upon metabolic stimulus exposure, which fails to inhibit Pparγ and its targets, driving abnormalities in lipid metabolism.
Lang, Z. [96]	2023	Liver Fibrosis	mice	GRh2	upregulation of IRF1	SLC7A11	-	GRh2 up-regulates IRF1 expression, resulting in the suppression of SLC7A11, which contributes to HSC ferroptosis and inactivation. GRh2 ameliorates liver fibrosis by enhancing HSC ferroptosis and inhibiting liver inflammation. GRh2 may be a promising drug for treating liver fibrosis.
Oh, J.E. [97]	2017	Liver Fibrosis	HSCs	IFN-γ and 1-methyl-L-tryptophan (1-MT)	upregulation of IRF1	-	-	IDO expression was markedly increased by IFN-γ through STAT1 activation and resulted in the depletion of tryptophan. This depletion induced G1 cell cycle arrest. When the cells were released from an IFN-γ-mediated G1 cell cycle arrest by treatment with 1-MT, the apoptosis of the HSCs was markedly increased through the induction of IFN-γRβ, IRF1, and FAS.
Iracheta-Vellve, A. [98]	2016	Liver Fibrosis	mice	STING	phosphorylation of IRF3	Caspase 3	-	In CCl4-treated hepatocytes, ER stresses results of the phosphorylation of TBK1 via STING, followed by phosphorylation of IRF3. IRF3 associates with BAX in the mitochondria through its BH3-only domain, leading to pro-apoptotic caspase 3 activation and hepatocyte apoptosis. After chronic CCl4 administration, hepatocyte apoptosis is associated with secondary necrosis, which results in liver fibrosis.
Yu, J.H. [99]	2024	Liver Fibrosis	mice	Gαs-coupled GPCR signaling	IRF3 phosphorylation	IL-33	GPCR-IRF3-IL-33	Gαs-coupled GPCR signaling increases IRF3 phosphorylation through cAMP-mediated activation of PKA. This leads to an increase in IL-33 expression, which further contributes to HSC activation. Hepatocyte GPCR signaling regulates IRF3 to control HSC trans-differentiation and provides insight for understanding the complex intercellular communication during liver fibrosis progression and suggests therapeutic opportunities for the disease.
Wu, Q. [100]	2024	Liver Fibrosis	mice	STING	IRF3 phosphorylation	CDK4/6	STING-IRF3-RB	The IRF3-RB interaction attenuates cyclin-dependent kinase 4/6 (CDK4/6)-mediated RB hyperphosphorylation that mobilizes RB to deactivate E2 family (E2F) transcription factors, thereby driving cells into senescence. STING-IRF3-RB signaling plays a notable role in HSCs within various murine models, pushing activated HSCs toward senescence. IRF3 global knockout or conditional deletion in HSCs aggravates liver fibrosis, a process mitigated by the CDK4/6 inhibitor.
Alzaid, F. [56]	2016	Liver Fibrosis	mice	hepatocellular stress	upregulation of IRF5	FasL, TNF, MHC II, IL6, and IL1β	-	IRF5-competent liver macrophages undergo proinflammatory activation in response to hepatocellular stress. Proinflammatory activation (M1) induces inflammatory a cytokine and death effector release that induces hepatocyte caspase (Casp)-dependent apoptosis. Activated factors include Fas ligand (FasL), TNF, MHC II, IL6, and IL1β. Proinflammatory activation induces type 1 and type 17 responses from CD4^+^T cells. Type 1 and 17 cytokines include IL17 and IFNγ. In IRF5-deficient macrophages, hepatocellular stress leads to immunosuppressive (MReg) polarization and secretion of IL10 and TGFβ. TGFβ expression induces differentiation of CD4+T cells into CD4^+^ FoxP3^+^ T cells, which contributes to IL-10 release. Secretion of IL-10 in IRF5 deficiency promotes anti-apoptotic signaling in hepatocytes mediated by B cell lymphoma 2 (BCL2) family members. This process maintains cell survival under stress.
Yu, M. [101]	2017	Hepatocellular Carcinoma	HCC cells	miR-345	deregulation of IRF1	Slug, Snail and Twist	mTOR/STAT3/AKT	Over-expression of *IRF1* mRNA was inversely correlated with the level of miR-345 in HCC specimens. Restoration of IRF1 resulted in promoted EMT and cell mobility in miR-345 overexpressing HCCLM3 cells. miR-345 acts as an inhibitor of the EMT process in HCC cells by targeting IRF1 and this study highlights the potential effects of miR-345 on prognosis and treatment of HCC.
Wang, Z. [102]	2023	Hepatocellular Carcinoma	HCC cells	SUMOylated IL-33	stabilized IRF1	PD-L1, IL-8, CXCL1	-	An increase in SUMOylated IL-33 in HCC cells in cocultures and in vivo-stabilized IRF1 and increased PD-L1 abundance and chemokine IL-8 secretion, which prevented the activation of cytotoxic T cells and promoted the M2 polarization of macrophages, respectively.
Yu, W. [103]	2023	Hepatocellular Carcinoma	NK cell	NR4A1	deregulation of IRF1	-	IFN-γ/p-STAT1/IRF1	NR4A1 was significantly highly expressed in tumor-infiltrating NK cells, which mediated the dysfunction of tumor-infiltrating NK cells by regulating the IFN-γ/p-STAT1/IRF1 signaling pathway, attenuated the anti-tumor function of NK cells, and reduced the efficacy of anti-PD-1 therapy.
Li, X. [104]	2023	Hepatocellular Carcinoma	HCC cells	CHK1	deregulation of IRF1	MICA	IRF1-MICA	Overexpressed CHK1 suppresses IRF1 expression through proteolysis. Cisplatin and CHK1 inhibition upregulate MICA expression through IRF1-mediated transcriptional effects. DNA damage regulates the interaction of CHK1 and IRF1 to activate anti-tumor immunity via the IRF1-MICA pathway in HCC.
Cui, X. [105]	2023	Hepatocellular Carcinoma	mice	FOXO1	upregulation of IRF1	CD206, IL-6, NO	-	These effects may be partially dependent on FOXO1 transcriptionally modulating the IRF-1/NO axis, exerting effects on macrophages and decreasing IL-6 release from macrophages in the tumor microenvironment indirectly. This feedback suppressed the progression of HCC by inactivating IL-6/STAT3 in HCC.
Yan, Y. [106]	2021	Hepatocellular Carcinoma	HCC cells	IFN-γ	upregulation of IRF4	miR-195, CHK1	-	IRF1 induces miR-195 to suppress CHK1 protein expression, which induces apoptosis of HCC cells. IRF1 expression or CHK1 inhibition also upregulates PD-L1 expression via increased STAT3 phosphorylation.
Yan, Y. [29]	2021	Hepatocellular Carcinoma	mice	-	upregulation of IRF1	CXCL10/CXCR3	IRF1/CXCL10/CXCR3	IRF1 increased CD8^+^ T cells, NK and NKT cells migration, and activated IFN-γ secretion in NK and NKT cells to induce tumor apoptosis through the CXCL10/CXCR3 paracrine axis.
Dong, K. [107]	2020	Hepatocellular Carcinoma	HCC cells	miR-301a	deregulation of IRF1	-	-	Chronic hypoxia induces miR-301a in HCC in vitro and decreases IRF1 expression. Finally, miR-301a inhibition increases apoptosis and decreases HCC cell proliferation.
Wan, P.Q. [108]	2020	Hepatocellular Carcinoma	HCC cells	miR-31	deregulation of IRF1	-	-	IRF1 was negatively correlated with miR-31 in HCC tissues and paired adjacent tissues. The expression level of miR-31 was inversely correlated with IRF1. MiR-31 inhibitor up-regulated the expression levels of IRF-1 in HuH7 cells, whereas the miR-31 mimic down-regulated the expression levels of IRF1.
Yan, Y. [109]	2016	Hepatocellular Carcinoma	HCC cells	miR-23a	deregulation of IRF1	-	-	MiR-23a mimics down-regulated IFNγ-induced IRF1 protein expression, while the miR-23a inhibitor increased IRF1. MiR-23a promotes HCC growth by downregulating IRF1.
Kim, G.W. [110]	2021	Hepatocellular Carcinoma	HCC cells	PTEN	negative phosphorylation of IRF3	PI3K/AKT	-	PTEN controlled IRF3 nuclear localization by negative phosphorylation of IRF3 at Ser97, and PTEN reduced carcinogenesis by inhibiting the PI3K/AKT pathway.
Yuan, J. [48]	2022	Hepatocellular Carcinoma	HCC cells	-	upregulation of IRF4	JAK2/STAT3	-	In vitro experiments demonstrated that the overexpression of IRF4 inhibited the proliferation and migration capacity of HCC cells by restricting the JAK2/STAT3 signaling pathway and epithelial-mesenchymal transition.
Cevik, O. [111]	2017	Hepatocellular Carcinoma	HCC cells	-	upregulation of IRF5	HCV protein translation and RNA replication	-	IRF5 re-expression inhibited HCV protein translation and RNA replication. IRF5 re-expression induced apoptosis via loss in mitochondrial membrane potential, down-regulated autophagy, and inhibited hepatocyte cell migration/invasion.
Fang, Y. [112]	2023	Hepatocellular Carcinoma	HCC cells	TRIM35	deregulation of IRF5	lactate dehydrogenase A (LDHA)	-	IRF5 was found to upregulate the expression of lactate dehydrogenase A (LDHA) and promote glycolysis. Tripartite motif containing 35 (TRIM35) interacted with IRF5, promoting its ubiquitination and degradation. These findings reveal the oncogenic function of IRF5 in the progression of HCC by enhancing glycolysis, further supporting the potential of IRF5 as a viable target for HCC therapy.
Wu, H. [66]	2022	Hepatocellular Carcinoma	mice	-	upregulation of IRF8	CCL20	-	Overexpression of IRF8 in HCC cells significantly enhanced antitumor effects in immune-competent mice, modulating infiltration of tumor-associated macrophages (TAMs) and T cell exhaustion in tumor microenvironment. IRF8 regulated recruitment of TAMs by inhibiting the expression of CCL20. Adeno-associated virus 8-mediated hepatic IRF8 rescue significantly suppressed HCC progression and enhanced the response to anti-PD-1 therapy.
Feng, L. [113]	2023	Hepatocellular Carcinoma	HCC cells	miR-424-3p	deregulation of IRF9	ISG15, IFITM1, OASL, TRIM21	SRF-STAT1/2/IRF9	miR-424-3p reduces the interferon pathway by attenuating the transactivation of SRF on STAT1/2 and IRF9 genes, which in turn enhances the matrix metalloproteinases (MMPs)-mediated ECM remodeling.

## Data Availability

No new experimental data were created.
